# Laminin and Matrix metalloproteinase 11 regulate Fibronectin levels in the zebrafish myotendinous junction

**DOI:** 10.1186/s13395-016-0089-3

**Published:** 2016-05-02

**Authors:** Molly H. Jenkins, Sarah S. Alrowaished, Michelle F. Goody, Bryan D. Crawford, Clarissa A. Henry

**Affiliations:** School of Biology and Ecology, University of Maine, 217 Hitchner Hall, Orono, ME 04469 USA; Department of Biology, University of New Brunswick, Fredericton, NB Canada; Graduate School of Biomedical Sciences and Engineering, University of Maine, Orono, ME 04469 USA; Present Address: Minerva Biotechnologies, Waltham, MA 02451 USA

**Keywords:** Zebrafish, Muscle development, Mmp11, Fibronectin, Laminin

## Abstract

**Background:**

Remodeling of the extracellular matrix (ECM) regulates cell adhesion as well as signaling between cells and their microenvironment. Despite the importance of tightly regulated ECM remodeling for normal muscle development and function, mechanisms underlying ECM remodeling in vivo remain elusive. One excellent paradigm in which to study ECM remodeling in vivo is morphogenesis of the myotendinous junction (MTJ) during zebrafish skeletal muscle development. During MTJ development, there are dramatic shifts in the primary components comprising the MTJ matrix. One such shift involves the replacement of Fibronectin (Fn)-rich matrix, which is essential for both somite and early muscle development, with laminin-rich matrix essential for normal function of the myotome. Here, we investigate the mechanism underlying this transition.

**Results:**

We show that laminin polymerization indirectly promotes Fn downregulation at the MTJ, via a matrix metalloproteinase 11 (Mmp11)-dependent mechanism. Laminin deposition and organization is required for localization of Mmp11 to the MTJ, where Mmp11 is both necessary and sufficient for Fn downregulation in vivo. Furthermore, reduction of residual Mmp11 in *laminin* mutants promotes a Fn-rich MTJ that partially rescues skeletal muscle architecture.

**Conclusions:**

These results identify a mechanism for Fn downregulation at the MTJ, highlight crosstalk between laminin and Fn, and identify a new in vivo function for Mmp11. Taken together, our data demonstrate a novel signaling pathway mediating Fn downregulation. Our data revealing new regulatory mechanisms that guide ECM remodeling during morphogenesis in vivo may inform pathological conditions in which Fn is dysregulated.

## Background

The muscle extracellular matrix (ECM) (called the myomatrix) plays a critical role in muscle development, physiology, homeostasis, and disease. Adhesion of muscle fibers to their surrounding myomatrix is required for muscle development and function, as evidenced by congenital muscle diseases —including *Duchenne*, *Becker*, and *Merosin-deficient* muscular dystrophies—which result from mutations that disrupt adhesion of muscle fibers to the myomatrix. The myomatrix is also critical for muscle homeostasis because it bears much of the passive load [1, 2], and many clinical symptoms involving range of motion and stiffness are likely derived from changes to the myomatrix [3]. The macromolecular composition of the myomatrix changes during muscle development and regeneration [4–9]. These changes in matrix composition define the biochemical and biophysical properties of the myomatrix; yet, the mechanisms that mediate dynamic changes in myomatrix composition during development and regeneration are unknown. In this study, we focused on elucidating mechanisms that underlie regulation of the myomatrix during development because this knowledge could inform efforts to maintain muscle health and prevent muscle diseases.

Remodeling of the ECM at the nascent myotendinous junction (MTJ) during zebrafish skeletal muscle development provides an ideal paradigm with which to investigate dynamic changes in the extracellular milieu. In teleost fishes, the ECM-rich somite boundaries give rise to MTJs that separate segmentally reiterated myotomes [10]. Elegant experiments have recently provided insight into how the ECM protein Fibronectin (Fn) is first polymerized at somite boundaries. During somite formation, integrin alpha5 activation, clustering, and signaling through Rap1b promote Fn polymerization [11]. Fn polymerization is limited to nascent somite boundaries by Eph/Ephrin signaling [12], reviewed in [13]. Subsequent to somitogenesis, a laminin-containing basement membrane is enriched at MTJs and Fn is downregulated [4, 9]. The mechanism underlying this downregulation of Fn at MTJs is unknown.

Understanding the regulation of Fn levels in the myomatrix is particularly important because Fn can either positively or negatively affect skeletal muscle depending on the context. Fn is an interstitial matrix protein best known for its functions during branching morphogenesis and cell migration [14–21]. Fn is necessary for muscle development: muscle is disorganized in zebrafish with reduced levels of Fn [22]. Although disrupted mesoderm development in Fn-null mice precludes analysis of how Fn contributes to muscle organization [23], elongating myocytes in wild-type mice express Fn receptors and appear to attach to Fn in the intersegmental ECM [4]. This result suggests that Fn is important for mammalian muscle development as well. Fn is also critical for muscle regeneration: Fn in the stem cell niche is both necessary and sufficient for satellite cell expansion [5]. These data clearly indicate that Fn is necessary for muscle development and regeneration, perhaps by generating a supportive ECM microenvironment. However, Fn expression in these contexts is transient. Sustained Fn expression may contribute to pathology in aging and diseased muscle. Abnormal Fn deposition is a hallmark of fibrosis in cardiac and skeletal muscle [24–26]. The replacement of contractile muscle tissue by Fn-rich fibrotic material leads to diminished organ function. Therefore, it is critical to understand the mechanisms underlying both Fn assembly and downregulation during muscle development and regeneration.

We utilized the zebrafish MTJ, where the characteristic downregulation of Fn is documented but remains mechanistically unknown, to identify regulators of Fn levels in vivo. We found that Fn levels are elevated in *laminin* mutant embryos. This result suggests that laminin deposition is a permissive cue for Fn downregulation. As laminin is not known to be a protease, we hypothesized that laminin regulates a member of the matrix metalloproteinase (MMP) family. Collectively, MMPs are able to cleave all known ECM proteins. MMPs are tightly regulated during development and homeostasis, and changes in MMP expression occur in many diseases [27, 28]. We identified Mmp11 (also known as stromelysin-3) as both necessary and sufficient for Fn downregulation in vivo. Significantly, Mmp11 localization to the MTJ is regulated by laminin. Inhibition of the residual Mmp11 activity in *laminin* mutants increases Fn levels and improves, but does not completely rescue, muscle structure. These results suggest that although decreased downregulation of Fn can improve muscle structure, the specific biochemical and biophysical properties of laminin are critical for normal muscle development. Taken together, our results highlight a novel crosstalk between myomatrix proteins and identify a MMP that regulates Fn levels in vivo.

## Methods

### Zebrafish husbandry, mutant, and transgenic lines

Zebrafish embryos were collected from natural spawnings of adult fish kept at 28.5 °C on a 14-h light/10-h dark cycle and staged according to [29]. The following strains were used: AB, *Gup/laminin beta1*^*tg210*^, *Sly/laminin gamma1*^*ti263A*^ (a generous gift from the Tuebingen stock center), *laminin gamma1*^*wi390Tg*^, and *Tg(HSP:mmp11-EGFP)*. All embryos were grown in embryo rearing media (1X ERM), which contained methylene blue to prevent microbial growth. All protocols were conducted in accordance with the University of Maine Institutional Animal Care and Usage Committee’s guidelines, which do not require approval for the use of zebrafish 3 days old and younger.

### Morpholino and transgenic construct injections

Morpholinos (MOs) were obtained from Gene Tools, LLC (Philomath, OR, USA). All MOs were diluted in sterile water and injected with a MPPI-2 Pressure Injector from ASI. Embryos were injected at the one-cell stage in the yolk with approximately 3 nL of MO. The nucleotide sequences of the MOs are as follows: *mmp11a* MO is 5′-GAGACCCGCATCCTCCTCCTGTATC-3′, *mmp11b* MO is 5′-CATGATGACGGAGAGCCGCACGCAG-3’, and standard control MO is 5′-CCTCTTACCTCAGTTACAATTTATA-3′. *Mmp11a* and *mmp11b* MOs were mixed, and embryos were injected such that they received a final concentration of approximately 2.5 ng of each MO. For controls, 12 ng of the standard control MO was injected and did not elicit a phenotype (data not shown). The *laminin gamma1* (*lamc1*) MOs used have been previously described [30] and recapitulate the mutant phenotype. The *nrk2b* MOs were injected as previously described [31].

The Tol2Kit gateway system [32] was used to create the *Tg(HSP:mmp11-EGFP)* construct. The expected product of this construct was a fusion protein of Mmp11 and EGFP, driven by a heat shock promoter (HSP). Primers used to create the *mmp11a* middle entry vector were *mmp11a* F 5′-GGGGACAAGTTTGTACAAAAAAGCAGGCTAGCACTTCCCACTTCACTTTCAGC-3′ and *mmp11a* R 5′-TCTTCGGATGTAACATGCCAcACCCAGCTTTCTTGTACAAAGTGGTCCCC-3′. The PCR product was recombined with the donor vector pDONR221 from the Tol2Kit via BP clonase (Invitrogen). This plasmid was then recombined with p5E-hsp70l, p3E-EGFPpA, and pDestTol2pA2 from the Tol2Kit, via LR clonase (Invitrogen) to create the final *Tg(HSP:mmp11-EGFP)* construct. Linearized pCS2-TP plasmid (kindly provided by Koichi Kawakami) was used as a template to generate capped mRNA encoding Tol2 transposase by in vitro transcription using SP6 polymerase (Message Machine, Ambion). The *Tg(HSP:mmp11-EGFP)* construct was co-injected with mRNA encoding Tol2 transposase to generate transient transgenic embryos (tTg). tTg embryos were injected at the one-cell stage into the cell and, at the designated developmental time point, heat-shocked at 40 °C for 1 h. A stable *Tg(HSP:mmp11-EGFP)* line was also generated, and embryos collected from natural spawnings of this line were heat-shocked as above and used for experiments.

### RNA extraction, cDNA synthesis, and quantitative PCR

RNA was extracted from whole, pooled embryos at 26 hours post fertilization (hpf) using a RNeasy Mini Kit (Qiagen) per manufacturer’s instructions. cDNA (500 ng per reaction) was synthesized using BioRad’s iScript reagents per manufacturer’s instructions. Quantitative PCR was performed using 300 nM of each primer per reaction, 25 ng of cDNA per reaction, BioRad’s ssoAdvanced reagents, and a BioRad CFX96 C1000 Touch real-time PCR machine per manufacturer’s instructions. Fold changes in mRNA abundance were calculated using the 2^(−ΔΔCt) equation. Ct values for genes of interest were normalized to the appropriate beta-actin or gapdh Ct values, and then values for experimental groups were normalized to their respective controls. A value of +/− 1 indicates no change from the control level of mRNA expression. Results from 2–3 independent biological replicates were averaged, and error bars represent standard error of the mean between biological replicates. qPCR primer sequences: beta-actin forward: 5′-TCGTGACCTGACAGACTACCTGAT-3′; beta-actin reverse: 5′-CGGACAATTTCTCCTTCGGCTGTG-3′; gapdh forward: TGGGCCCATGAAAGGAAT; gapdh reverse: ACCAGCGTCAAAGATGGATG; Fn1a forward: CCACCCACTGATCTGAACCT; Fn1a reverse: TCACTCTGTAGCCCGTGATG; Fn1b forward: ATTCAACGCACGTTCCTACC; Fn1b reverse: TAATTTTGCCCTTGCCTGAC; lama1 forward: TGCTGGAGCTCATCAACAAC; lama1 reverse: TTTTCCAGCACAGACACTGC; lama2 forward: GCAGAAATCCTGGATGTGGT; lama2 reverse: TGGAGGTGGGATTCTCCATA; lamb1 forward: GCAGCTCAAAAAGGATCTGG; lamb1 reverse: AAGTTTCTCGCTGGCCTGTA; and lamc1 forward: GGTTGCAAACCATGTGACTG; lamc1 reverse: CAAATTCTGCAGGTCAAGCA.

### Protein immunoblots

Protein for immunoblots was prepared from whole zebrafish embryos at 23, 26, 32, or 48 hpf) using the method described by [33]. Protein was resolved on a 10 % SDS-PAGE gel, transferred to PVDF membrane, blocked in 5 % dry milk in phosphate buffered saline with 0.1 % Tween20 (PBST), incubated in 1° antibody (anti-Fibronectin 1:500, Sigma; anti-Mmp11 1:500, Anaspec; anti-α-tubulin 1:1000, Sigma) at 4 °C overnight. The membrane was then washed in PBST, incubated in 2° antibody (anti-rabbit HRP 1:2000, Pierce; anti-mouse HRP 1:2000, Pierce), washed, detected with Supersignal West Dura (Pierce), and imaged on a CCD LAS 4000 Fuji camera. Relative protein amounts were quantified in ImageJ and normalized to loading controls (when present).

### Fixation/phalloidin staining/immunocytochemistry

Dechorionated embryos were fixed in 4 % paraformaldehyde (PFA) for 4 h at room temperature (RT) or overnight at 4 °C, and fix was removed by washing five times for 5 min each in 0.1 % PBS-Tween20.

Alexa Fluor 546 phalloidin (Molecular Probes) staining required permeabilizing fixed embryos for 1.5 h in 2 % PBS-Triton and then incubating in phalloidin (1:20 in 2 % PBS-Triton) for 1–4 h at RT or overnight at 4 °C. Embryos were then deyolked and imaged, or subsequent antibody staining was performed.

For general antibody staining, fixed embryos were blocked for at least 1 h at RT and incubated overnight at 4 °C in primary antibody (polyclonal anti-laminin-111 1:50 (Sigma); polyclonal anti-Fibronectin generated against human Fn 1:50 (Sigma); anti-F59 1:10 (DSHB); anti-β-dystroglycan 1:50). All antibodies were diluted in block (5 % *w*/*v* bovine serum albumin (BSA) in PBS with 0.1 % Tween20). Embryos were washed at RT 2–4 h in block then incubated in secondary antibody overnight at 4 °C or for 4 h at RT (Alexa-Flour 488, 546, 633 conjugated goat anti-mouse or goat anti-rabbit secondary antibodies 1:200 (Invitrogen)). Embryos were then washed for 1 h in 0.1 % PBS-Tween20.

Anti-Mmp11 antibody staining involved fixing embryos overnight at 4 °C in Dent’s fixative (80 % MeOH, 20 % DMSO). Embryos were then washed three times for 15 min each in PBS with 0.1 % Tween20, blocked for 4 h at RT, and incubated in an antibody raised against zebrafish Mmp11 antigen (Anaspec, 1:50) overnight at 4 °C. Embryos were washed three times for 15 min each and then incubated in goat anti-rabbit secondary (Invitrogen, 1:200) overnight at 4 °C. Embryos were washed, deyolked, mounted, and imaged.

### Imaging

Brightfield images of live embryos were taken on the Zeiss SteREO Discovery V12 microscope at ×25 or the Zeiss Axio Imager Z1 microscope with the ×5 objective. Live embryos were anesthetized using a non-lethal dose of MS-222. Fixed embryos were deyolked in PBS, side-mounted in 80 % glycerol/20 % PBS, and imaged with the ×20 objective of a Zeiss Axio Imager Z1 microscope with a Zeiss ApoTome attachment and optimized by averaging five frames. For images where fluorescence levels were to be compared, exposure times were kept constant throughout the imaging of that experiment. All image modifications were performed in Adobe Photoshop with Gaussian filtering (0.3 pixel) and unsharp mask (50 %, 1 pixel) prior to being collated in Adobe Illustrator.

### Histograms

Images were imported into ImageJ software, and the intensity profiles were plotted. The resulting data were analyzed statistically using Microsoft Office Excel software. We calculated the maximum intensity, and all values were normalized to it. The percentage of maximum intensity was plotted.

## Results

### Reciprocal expression of laminin and Fn at the fast-twitch muscle MTJ during muscle development

**Fig. 1:**
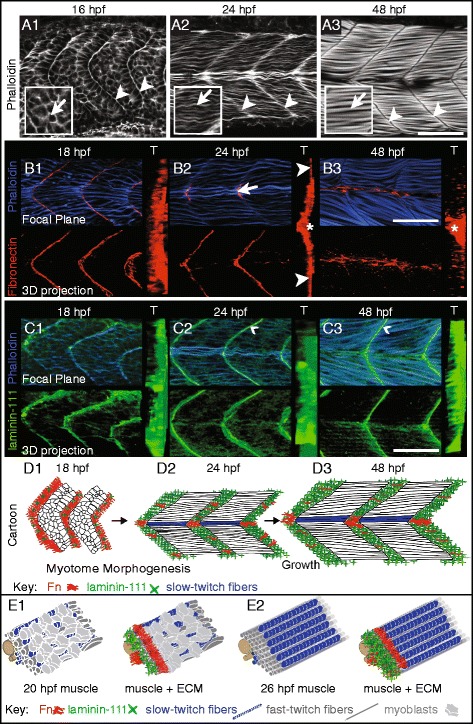
Fn and laminin are reciprocally expressed at the MTJ adjacent to fast-twitch fibers during muscle morphogenesis. **A**–**C** Anterior left, dorsal top, side-mounted, wild-type embryos. Phalloidin staining for actin to visualize the cell morphological changes during muscle morphogenesis. **A1** At 16 hpf, muscle precursors are round (*arrow* in *inset*) and actin accumulates between somite boundaries (*arrowheads*). **A2** At 24 hpf, muscle cells have elongated (*arrow* in *inset*) and actin continues to accumulate at the boundary (*arrowheads*). **A3** At 48 hpf, muscle fibers have undergone further growth and become striated (*arrow* in *inset*). MTJs are devoid of actin staining (*arrowheads*). **B1**–**B3** Focal planes, 3D projections (anterior left, dorsal top, side-mounted), and transverse views ((*T*) lateral right, dorsal top) of phalloidin (*blue*) and Fn antibody staining (*red*). *b1* At 18 hpf, Fn accumulates throughout the medial-lateral extent of the MTJ. **B2** At 24 hpf, Fn is adjacent to lateral, superficial slow-twitch fibers (*arrowheads* in *T*) as well as medial MPs (*arrow* in focal plane and *asterisk* in *T*). **B3** At 48 hpf, Fn is downregulated throughout the medial portion of the MTJ except adjacent to MPs (*asterisk* in *T*). **C1**–**C3** Focal planes, 3D projections (anterior left, dorsal top, side-mounted), and transverse views ((*T*) lateral right, dorsal top) of phalloidin (*blue*) and laminin-111 antibody staining (*green*). **C1** At 18 hpf, laminin-111 begins to polymerize at the MTJ. **C2** At 24 hpf, polymerized laminin-111 is adjacent to slow- and fast-twitch muscle fibers (*arrowhead* in focal plane points to laminin-111 adjacent to fast-twitch muscle fibers). **C3** At 48 hpf, laminin-111 remains throughout the medial-lateral extent of the MTJ (*arrowhead* denotes laminin-111 adjacent to fast-twitch muscle fibers). **D**, **E** Models of Fn-laminin dynamics at the MTJ in a medial focal plane (**D**) and a 3D rendering (**E**) over developmental time. The presence of slow-twitch fibers (*blue*) along the midline confirms that focal planes are medial sections. **D1** At 18 hpf, a mainly Fn myomatrix segregates somites. **D2**–**D3** In medial focal planes at 24 and 48 hpf, because slow-twitch fibers have migrated laterally, a mainly laminin-111 myomatrix separates myotomes. **E1** As slow-twitch fibers migrate laterally, myoblasts elongate and the mainly Fn matrix is replaced by a mainly laminin-111 matrix medial to the location of migrating slow-twitch fibers. **E2** After slow-twitch fiber migration is complete, the MTJ is primarily laminin-111 adjacent to fast-twitch fibers while Fn remains adjacent to slow-twitch fibers. *Scale bars* are 50 μm

During axial skeletal muscle development, somites (blocks of mesodermal cells) transform into myotomes (groups of muscle fibers). Initially short myoblasts (Fig. 1 A1 inset, arrow) undergo elongation (Fig. 1 A2–A3 insets, arrows) and attach to MTJs (Fig. 1 A2–A3), arrowheads) that are derived from initial somite boundaries (Fig. 1 A1 , arrowheads). Slow- and fast-twitch muscle fibers are spatially segregated during zebrafish development [34]. Slow-twitch muscle fibers are specified medially and then migrate laterally. Slow-twitch muscle fiber migration triggers the elongation of fast-twitch muscle fibers [35]. These morphological changes are accompanied by molecular changes in the ECM: laminin increases and Fn is downregulated [9]. At 18 hpf, in segments where slow-twitch muscle migration has not yet occurred, Fn is present throughout the medial-lateral extent of the MTJ (Fig. 1B1, see 3D projection and transverse view (T)). Fn is then downregulated medial to slow-twitch muscle fibers as they migrate laterally. In other words, Fn is downregulated at the MTJ adjacent to fast-twitch fibers after slow-twitch fibers migrate through the fast-twitch domain. This downregulation results in Fn concentrating adjacent to slow-twitch but not fast-twitch muscle fibers at 24 hpf (Fig. 1B2, arrow). Thus, migrating slow-twitch muscle fibers provide a spatial and temporal marker for normal Fn downregulation. By 48 hpf, Fn is detected at relatively low levels throughout the MTJ. Fn concentrates at a subset of slow-twitch muscle fibers called muscle pioneers (MPs) (Fig. 1B3, asterisk in T). The pattern of laminin-111 accumulation at the MTJ adjacent to fast-twitch fibers is reciprocal to that of Fn. Laminin-111 deposition at the MTJ increases through 48 hpf (Fig. 1 C1–C3, arrowheads in Fig. 1 C2, C3). The changes in muscle cell morphogenesis, Fn, and laminin-111 at the MTJ are cartooned in Fig. 1D (side view) and Fig. 1E (oblique transverse view).

### Laminin organization is required for Fn downregulation at the MTJ

**Fig. 2:**
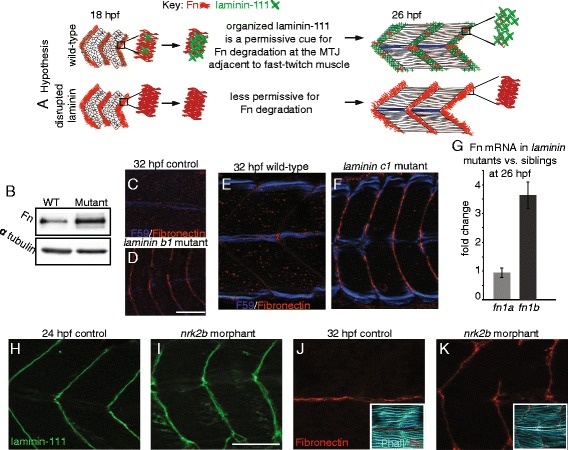
Organized laminin deposition is required for Fn downregulation. **a** Hypothesis that laminin deposition regulates Fn downregulation. *Upper panel*: laminin is polymerized at the MTJ and Fn is downregulated. *Lower panel*: laminin deposition/organization is disrupted and Fn perdures at the MTJ. **b** Western blot of whole embryo homogenates at 48 hpf using anti-Fn antibody. *Laminin gamma1* mutants have approximately 2.5-fold more Fn compared to siblings. Alpha-tubulin was used as a loading control. (*C*–*F*; *H*–*K*) Anterior left, dorsal top, side-mounted embryos. Fn antibody (*red*), F59 antibody to label slow-twitch fibers (*dark blue*), laminin-111 antibody (*green*), phalloidin staining (*light blue*). **c** Medial focal plane of 32 hpf sibling. Note Fn (*red*) has been downregulated adjacent to fast-twitch fibers. **d** Medial focal plane of 32 hpf *laminin beta1* mutant (*n* = 16 embryos). Note that Fn persists adjacent to medial, fast-twitch fibers. **e** Medial focal plane of 32 hpf sibling. Note Fn (*red*) has been downregulated adjacent to fast-twitch fibers. **f** Medial focal plane of 32 hpf *laminin gamma1* mutant (*n* = 28 embryos). Note that Fn persists adjacent to medial, fast-twitch fibers in *laminin beta1* and *gamma1* mutant zebrafish. **g** Relative mRNA abundance of *fn1a* and *fn1b* in *laminin gamma1* mutants compared to siblings at 26 hpf (*n* = 3 biological replicates of at least five embryos each). *Fn1a* expression is unchanged whereas *fn1b* expression is upregulated in *laminin gamma1* mutants. **h** Focal plane of laminin-111 antibody staining in a 24-hpf control embryo. **i** Focal plane of laminin-111 antibody staining in a 24-hpf *nrk2b* morphant embryo. Note that laminin-111 protein is present, but that the organization of laminin-111 at MTJs is disrupted. **j** Medial focal plane of Fn antibody staining in a 32-hpf control embryo. **k** Medial focal plane of Fn antibody staining in a 32-hpf *nrk2b* morphant embryo (*n* = 9 embryos). Fn is present at the MTJ adjacent to MPs and fast-twitch fibers in *nrk2b* morphants. *Scale bars* are 50 μm

The reciprocal regulation of Fn and laminin in the fast-twitch muscle domain suggests the hypothesis that laminin organization/deposition/signaling acts as a cue/checkpoint that is permissive for Fn downregulation (Fig. 2a). If this is the case, absence of laminin at the MTJ would result in increased levels of Fn. Laminins are cross-shaped heterotrimeric proteins comprised of an alpha, beta, and gamma chain. Translated laminin gene products (one alpha, one beta, and one gamma) self-assemble into cross-shaped laminin proteins with the alpha subunit constituting the long arm of the cross and the beta and gamma subunits forming the short arms of the cross [36, 37]. Assembled laminin proteins then polymerize with other laminin proteins and other ECM molecules to form specialized basement membranes. Laminin-111 (assembled from laminin alpha1, beta1, and gamma1 gene products) is the developmental isoform of laminin relevant for early muscle development [30, 38, 39]. We tested the hypothesis that laminin organization/deposition/signaling acts as a cue/checkpoint that is permissive for Fn downregulation by assessing Fn protein levels in *laminin beta1* or *laminin gamma1* mutant zebrafish (Fig. 2a). As *laminin beta1* and *laminin gamma1* gene products are both part of the assembled laminin-111 protein, neither of these mutant zebrafish lines will have the laminin-111 isoform or any other isoform of laminin that utilizes *laminin beta1* or *laminin gamma1* gene products, respectively. Protein immunoblots of whole embryo extracts at 48 hpf showed increased Fn levels in *laminin gamma1* mutants compared to siblings (Fig. 2b). Slow-twitch fiber migration is slightly delayed in *laminin beta1* and *gamma1* mutant embryos but recovers by 26 hpf [40]. Thus, at 32 hpf, slow-twitch fibers can be used as a marker for Fn: Fn should be downregulated medial to slow-twitch fibers. Fn was abnormally abundant throughout the medial-lateral extent of the MTJ in *laminin beta1* or *laminin gamma1* mutants compared to siblings at 32 hpf (compare Fig. 2D to C and Fig. 2F to E). To test whether laminin regulates Fn at the transcriptional level, we assayed *fn1a* and *fn1b* mRNA abundance in *laminin gamma1* mutants and their siblings at 26 hpf. *Fn1a* transcript abundance was not changed in *laminin gamma1* mutants compared to sibling controls; however, *fn1b* transcript abundance was approximately fourfold higher in *laminin gamma1* mutants compared to controls (Fig. 2g, *fn1a*: 0.9 +/− 0.2 or 0.9 +/− 0.1 in *laminin gamma1* mutants relative to controls normalized to *beta-actin* or *gapdh*, respectively; *fn1b*: 3.6 +/− 0.5 or 4.1 +/− 0.5 in *laminin gamma1* mutants relative to controls normalized to *beta-actin* or *gapdh*, respectively). The fact that multiple assays—qPCR, Western blotting, and immunocytochemistry—show increased Fn in *laminin* mutant embryos supports the hypothesis that laminin is required for Fn downregulation.

The phenotype of *laminin beta1* or *laminin gamma1* mutants is fairly severe: embryos are truncated, somites are much narrower in the anterior-posterior axis, and muscle fiber elongation is delayed [41]. We previously showed that Nicotinamide Riboside Kinase 2b (Nrk2b) is required for organized laminin at MTJs during muscle development [31]. The phenotype of *nrk2b* morphant embryos is milder than that of *laminin beta1* or *laminin gamma1* mutants: embryos are longer, somites are wider, and neither development nor muscle fiber elongation is delayed. We used *nrk2b* morphants to ask whether Fn remains at MTJs in a situation where laminin protein is present, but the organization of laminin-111 at MTJs is disrupted compared to controls (Fig. 2h, i). In medial focal planes of control embryos at 32 hpf, Fn was concentrated at the MTJ adjacent to MPs and the horizontal myoseptum and was not visible at the MTJ adjacent to fast-twitch muscle fibers (Fig. 2j). Fn was also concentrated at the MTJ adjacent to medial MPs in *nrk2b* morphant embryos at 32 hpf; however, Fn concentration at the MTJ adjacent to fast-twitch muscle fibers was much higher in *nrk2b* morphant embryos compared to controls at 32 hpf (Fig. 2k). As stated above, the relative abundance of *fn1b* transcripts was upregulated in in *laminin gamma1* mutants. The relative abundance of *fn1b* is similarly upregulated in *nrk2b* morphants compared to controls (*fn1b*: 2.9 +/− 0.6 or 3.3 +/− 1.2 in *nrk2b* morphants relative to controls normalized to *beta-actin* or *gapdh*, respectively). Taken together, the above data indicate that organized laminin, and not just the presence of laminin protein, is necessary for Fn downregulation during MTJ development.

### Laminin is necessary to recruit/retain Mmp11 at the MTJ

**Fig. 3:**
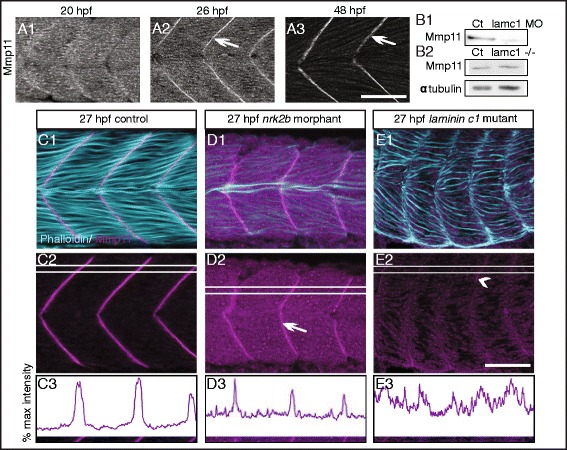
Normal laminin deposition/organization is necessary for concentration of Mmp11 at MTJs. **A1**–**A3** Anterior left, dorsal top, side-mounted, wild-type embryos stained with anti-Mmp11 antibody at 20, 26, or 48 hpf, respectively. Note that Mmp11 increasingly concentrates at MTJs (*white arrows*) over developmental time. **B1**, **B2** Western blots of whole embryo homogenates at 26 hpf using an anti-Mmp11 antibody. **B1** *Laminin gamma1* morphants have approximately 8.5-fold less Mmp11 protein compared to controls. **B2** *Laminin gamma1* mutants show no change (approximately 1.2-fold more) Mmp11 protein compared to siblings. (**C**–**E**) Side view, anterior left, dorsal top, 27 hpf embryos stained with phalloidin (*light blue*), and anti-Mmp11 antibody (*purple*). **C1**–**C3** Control embryo. Mmp11 localizes to the MTJ (peaks in **C3**) and is absent from the myotome (valleys in *c3*). *c3* Histogram of relative pixel intensities across the white rectangle in panel *c2. d1*–*d3* *nrk2b* morphant. Mmp11 is expressed and concentrates at MTJ in *nrk2b* morphants (*white arrow* in *d2* and peaks in *d3*); however, Mmp11 localization is disrupted as can be seen by the increased staining within myotomes (higher intensity of valleys in *d3* compared to *c3*) (*n* = 13 embryos). *d3* Histogram of relative pixel intensities across the white rectangle in panel **D2. E1**–**E3** *laminin gamma1* mutant. Mmp11 does not localize to MTJs (absence of clear peaks and valleys in **E3**), except in small patches (*white arrowhead* in **E2**) (*n* = 7 embryos). **E3** Histogram of relative pixel intensities across the white rectangle in panel **E2**. *Scale bars* are 50 μm

Our data show that increased transcription of *fn1b* (Fig. 2g) could explain the persistence of Fn protein at MTJs in *laminin* mutants; however, the low level of expression of *fn1a* and *fn1b* mRNA after segmentation is complete [21, 42] also suggests the possibility that changes in Fn protein levels at the MTJ may be mediated posttranslationally. Although laminin is necessary for normal Fn downregulation, laminin itself is not a candidate for degrading Fn because laminin has not been shown to have protease activity. Collectively, the members of the MMP family of proteases are able to degrade all proteins found in the ECM [43]. We therefore looked for MMPs that fit two criteria: (1) MMP “X” would need to be expressed at the right time and place to play a role in Fn degradation at the MTJ, and (2) laminin would need to regulate MMP “X.” The rationale for the second criteria is that if laminin is required for Fn downregulation, one potential mechanism would be that laminin regulates MMP “X” expression, localization, or activity. Mmp11 (stromelysin-3) is strongly expressed at the MTJ in zebrafish at 2 days post fertilization [44]. We analyzed Mmp11 expression earlier in muscle development and found that Mmp11 was initially distributed throughout the somitic mesoderm (Fig. 3A1). Mmp11 was more concentrated at the MTJ at 26 hpf (Fig. 3A2) and was robustly localized to the MTJ at 48 hpf (Fig. 3A3). Thus, Mmp11 is expressed in the right time and place to play a role in Fn downregulation.

We next asked if laminin deposition/organization is necessary for Mmp11 expression by analyzing Mmp11 protein levels in laminin-deficient embryos. Mmp11 protein levels were decreased in whole embryo homogenates of *laminin gamma1* morphant embryos compared to controls at 26 hpf (Fig. 3B1); however, overall Mmp11 protein levels were unchanged in whole embryo homogenates of 26 hpf *laminin gamma1* mutants compared to siblings (Fig. 3B2). This discrepancy could be due to the function of maternally provided laminin gamma1 in the mutants. *Laminin gamma1* is maternally expressed [30]. The phenotype of morphants is sometimes more severe than mutants because morpholinos can block translation of both maternal and zygotic transcripts [45]. Therefore, *laminin gamma1* morphants and mutants would both lack zygotic laminin gamma1 protein. In contrast, mutants would express maternal laminin gamma1 protein but morphants would not. The reduction in Mmp11 levels in *laminin gamma1* morphants could be a non-specific artifact of morpholino knockdown technology or the maternally supplied laminin gamma1 in *laminin gamma1* mutants could be sufficient for normal Mmp11 expression through primary muscle development.

As the effect of laminin on Mmp11 was not clear from Western analysis, we performed immunocytochemistry to visualize Mmp11 localization in embryos with disrupted laminin-111 at MTJs. Control, mutant, and morphant images were imaged with the same settings and post-processed identically with Photoshop. In control embryos at 27 hpf, Mmp11 was strongly concentrated at MTJs (Fig. 3 C1, C2). A histogram of relative pixel intensities across the white rectangle in Fig. 3C2 corroborates that Mmp11 localized to MTJs: tall, tight peaks of bright pixel intensities occurred at MTJs and low valleys between peaks represent dim pixel intensities throughout myotomes. We used *nrk2b* morphants, where laminin at the MTJ is significantly less organized, to ask whether laminin organization regulates Mmp11 localization. Mmp11 concentrated at the MTJ in *nrk2b* morphants (Fig. 3 D1, D2, white arrow); however, Mmp11 was also observed throughout the myotome (Fig. 3D2). In the histogram of relative pixel intensities, tall peaks of bright pixel intensities occurred at MTJs (Fig. 3D3), supporting the observation that Mmp11 localized to MTJs in *nrk2b* morphants. However, pixel intensities between peaks were higher in *nrk2b* morphants compared to controls (Fig. 3 C3, D3). This reflects the observation that more Mmp11 localized within the myotome in *nrk2b* morphants than controls. Mmp11 concentration at the MTJ was even more disrupted in *laminin gamma1* mutant embryos. Mmp11 only concentrated at MTJs in small patches (Fig. 3E2, arrowhead), and the histogram of relative pixel intensities showed wide, irregular peaks, and high pixel intensities within myotomes (Fig. 3(E3)). These data suggest that laminin adhesion and/or signaling modulate Mmp11 localization.

### Mmp11 is necessary and sufficient for Fn downregulation

**Fig. 4:**
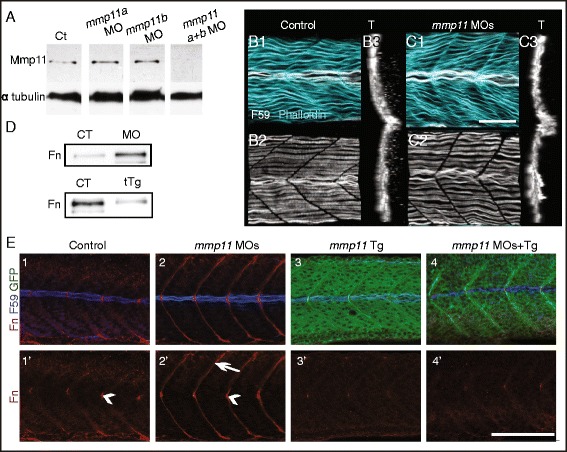
Mmp11 is necessary and sufficient for Fn downregulation. **A** Western blot of whole embryo homogenates at 26 hpf using Mmp11 antibody. Injection of individual MOs (a or b) alone did not reduce Mmp11 protein levels compared to controls. Co-injection of both MOs (a + b) reduced Mmp11 levels by approximately 14-fold compared to controls. Alpha-tubulin was used as a loading control. **B**, **C** Focal planes, 3D projections (anterior left, dorsal top, side-mounted), and transverse views ((*T*) lateral left, dorsal top) of control embryo (**B**) and *mmp11* morphant (**C**) at 27 hpf, fast muscle fibers are stained with phalloidin (*light blue*) and slow-twitch fibers are stained with F59 (*white*). **D** Western blot of whole embryo homogenates at 32 hpf (*upper panel*) or 23 hpf (*lower panel*) using anti-Fn antibody. *Upper panel*: Fn protein level is increased by approximately 4.7-fold in *mmp11* morphants compared to controls. *Lower panel*: Fn protein level is decreased by approximately 4.2-fold in embryos overexpressing *mmp11*-EGFP (tTg) compared to controls. **E** Medial focal plane, anterior left, dorsal top, side-mounted embryos at 26 hpf stained with Fn antibody (*red*) and F59 antibody (*blue*). Overexpression of *mmp11*-EGFP is *green*. **E1**, **E1’** Control. (*E2*–*E2’*) *mmp11* morphant (*n* > 60 embryos). Note that Fn concentrates adjacent to MPs in both controls and *mmp11* morphants (*arrowheads* in **E1’** and **E2’**) but only concentrates adjacent to fast-twitch muscle fibers in *mmp11* morphants (*arrow* **E2’**). **E3**–**E3’** *mmp11*-EGFP transgenic. Note that Fn is downregulated adjacent to MPs in transgenics compared to controls. **E4**–**E4’** *mmp11* morphant overexpressing *mmp11*-EGFP (*n* > 50 embryos). *Mmp11*-EGFP overexpression reduced Fn levels throughout the MTJ in *mmp11* morphants. *Scale bars* are 50 μm

The above data indicate that laminin is necessary for both Mmp11 localization to the MTJ and Fn downregulation at the MTJ. Therefore, Mmp11 cleavage of Fn could be a mechanism through which laminin promotes downregulation of Fn at the MTJ. However, MMP11 shows only weak protease activity against Fn in vitro [46]. As in vivo functions for Mmp11 are not fully elucidated, Mmp11 could directly or indirectly modulate Fn levels at the MTJ. We used a reverse genetics approach to knockdown Mmp11 protein levels and assay the effect on Fn. The human *MMP11* (*ST-3*) gene has two zebrafish paralogs, *mmp11a* and *mmp11b* [44], so we designed translation-blocking morpholinos (MOs) against both. Injection of either MO alone did not appreciably reduce Mmp11 expression on immunoblots of whole embryo homogenates; however, co-injection of both MOs dramatically decreased Mmp11 levels (Fig. 4A). We therefore co-injected *mmp11a* and *mmp11b* MOs in all subsequent experiments.

Injection of *mmp11* MOs did not overtly affect body, slow-twitch muscle, or fast-twitch muscle morphology compared to control embryos (Fig. 4B, C and data not shown). We used *mmp11* morphants to ask whether Mmp11 is required for Fn downregulation. We found Fn protein to be increased in whole embryo homogenates of *mmp11* morphants compared to controls (Fig. 4D). The increase in Fn was also apparent with immunostaining. In control embryos, Fn was downregulated medial to migrating slow-twitch muscle fibers, resulting in very little Fn at the MTJ adjacent to fast-twitch muscle fibers (Fig. 4E1, E1’). In contrast, Fn remained at the MTJ adjacent to slow-twitch and fast-twitch fibers in *mmp11* morphants (Fig. 4E2, E2’, arrowhead and arrow, respectively). Thus, multiple types of analysis—western blotting and whole mount immunocytochemistry—show increased Fn levels in *mmp11* morphants. The fact that embryonic development is grossly normal in *mmp11* morphant embryos suggests that the requirement of Mmp11 for Fn downregulation is specific and not due to confounding requirements for Mmp11 during early development. Taken together, these data suggest that Mmp11 is necessary for normal downregulation of Fn during muscle development.

We next asked whether Mmp11 is sufficient to downregulate Fn. We found that ubiquitous overexpression of Mmp11 during early development disrupted morphogenesis (data not shown). We therefore expressed Mmp11 under control of the heat shock promoter to allow for control over the timing of Mmp11 overexpression. Embryos were injected with the *Tg(HSP:mmp11-EGFP)* construct and *tol2 transposase* mRNA at the one-cell stage, grown at normal temperature, and then heat-shocked at 18 hpf. At 5 hours post-heat shock, transient overexpression of Mmp11 resulted in decreased levels of Fn compared to controls on immunoblots from whole embryo lysates (Fig. 4D). In embryos derived from matings of the stable *Tg(HSP:mmp11-EGFP)* line, Mmp11-EGFP expression was observed throughout the myotome and concentrated at MTJs (Fig. 4E3). Fn immunostaining revealed reduced Fn throughout the MTJ, even in the muscle pioneer domain, compared to controls (Fig. 4E3’). The effects of ectopic Mmp11 on Fn levels were even more pronounced in *mmp11* morphants. In *mmp11* morphants that express ectopic Mmp11-EGFP, Fn was drastically reduced at the MTJ compared to *mmp11* morphant controls (Fig. 4E4, E4’). These data indicate that Mmp11 overexpression is sufficient to downregulate Fn at the MTJ. Furthermore, as Mmp11 expression rescues the *mmp11* morphant phenotype, this experiment provides evidence that *mmp11* MOs are specific and suggests that the aberrant upregulation of Fn seen in *mmp11* morphants is not an off-target effect of *mmp11* MOs.

### Laminin functions upstream of Mmp11 in the regulation of Fn at MTJs

**Fig. 5:**
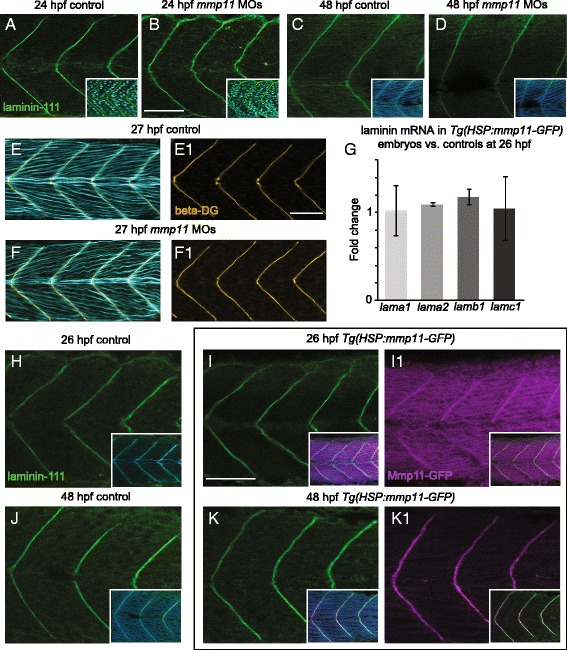
Laminin-111 at MTJs in not affected by Mmp11 knockdown or overexpression. **A**–**F1**, **H**–**K1** Anterior left, dorsal top, side-mounted embryos stained for laminin-111 (*green*), nuclei (*light blue*), actin (*light* or *dark blue*), beta-dystroglycan (*orange*), or showing Mmp11-GFP (*purple*). **A**–**B** laminin-111 antibody and nuclei staining in 26 hpf embryos. **A** Control embryo. **B** *mmp11* morphant embryo. **C**–**D** laminin-111 antibody and phalloidin staining in 48 hpf embryos. **C** Control embryo. **D** *mmp11* morphant embryo (*n* = 8 embryos). Note that laminin-111 appears normal in *mmp11* morphants compared to controls at 26 and 48 hpf. **E**–**F1** Beta-dystroglycan antibody and phalloidin staining in 27 hpf embryos. **E**, **E1** Control embryo showing merged panel **E** and beta-dystroglycan antibody staining alone (**E1**). **F**, **F1** *mmp11* morphant embryo showing merged panel **F** and beta-dystroglycan antibody staining alone (**F1**). Normal beta-dystroglycan antibody staining in *mmp11* morphants indirectly supports that laminin-111 is unaffected by Mmp11 knockdown. **G** Relative mRNA abundance of *lama1*, *lama2*, *lamb1*, and *lamc1* in *mmp11* morphants compared to controls at 26 hpf. The expression of the *laminin* genes assayed is unchanged by Mmp11-GFP overexpression (*n* = 2 biological replicates of at least five embryos each). **H**–**K1** laminin-111 antibody and phalloidin staining in heat-shocked AB controls and *Tg(HSP:mmp11-GFP)* embryos. **H** Control embryo at 26 hpf showing laminin-111 and laminin-111 merged with phalloidin (*inset*). **I**, **I1** 26 hpf *Tg(HSP:mmp11-GFP)* embryo showing laminin-111 (**I**), laminin-111 merged with phalloidin and Mmp11-GFP (*inset* in **I**), Mmp11-GFP (**I1**), and Mmp11-GFP merged with laminin-111 (*inset* in **I1**). **J** Control embryo at 48 hpf showing laminin-111 and laminin-111 merged with phalloidin (*inset*). **K**, **K1** 48 hpf *Tg(HSP:mmp11-GFP)* embryo showing laminin-111 (**K**), laminin-111 merged with phalloidin and Mmp11-GFP (*inset* in **K**), Mmp11-GFP (**K1**), and Mmp11-GFP merged with laminin-111 (*inset* in **K1**). Note that the timing of Mmp11-GFP MTJ localization recapitulates that of native Mmp11 protein, Mmp11-GFP and laminin-111 co-localize at MTJs, and laminin-111 appears normal in *Tg(HSP:mmp11-GFP)* embryos (*n* = 5 embryos). *Scale bars* are 50 μm

The data presented thus far fit a unidirectional model where laminin is necessary for localization of Mmp11 to MTJs, and Mmp11 is then necessary and sufficient for the regulation of Fn. As many regulatory networks involve bidirectional signaling interactions, it is also possible that Mmp11 regulates laminin and laminin, in turn, affects Fn levels at MTJs. To investigate this possibility, we analyzed laminin mRNA and protein localization in *mmp11* morphants and/or transgenics that overexpress Mmp11. Deposition of laminin-111 protein at MTJs in *mmp11* morphants was similar to controls at both 24 and 48 hpf (Fig. 5A–D). Furthermore, antibody staining for beta-dystroglycan (part of the dystrophin-glycoprotein complex receptor for laminin-111) in 27 hpf *mmp11* morphants compared to controls was also similar (Fig. 5E–F1). This result supports the observation that laminin-111 deposition is unaffected in *mmp11* morphants.

Next, we analyzed mRNA expression of *laminin* genes and laminin-111 protein localization in zebrafish stably overexpressing Mmp11-GFP. Expression of relevant laminin gene products for the isoforms of laminin expressed in skeletal muscle development and homeostasis (i.e., *laminin alpha1*, *alpha2*, *beta1*, *and gamma1*) was not changed in 26 hpf *Tg(HSP:mmp11-GFP)* embryos compared to heat-shocked AB controls (Fig. 5G). Additionally, laminin-111 deposition and localization at MTJs appeared normal at 26 and 48 hpf in embryos overexpressing Mmp11-GFP (Fig. 5H, I and Fig. 5J, K, respectively). The expression and localization of Mmp11-GFP recapitulated that of the endogenous protein: Mmp11-GFP began to localize to MTJs at 26 hpf and was robustly localized to MTJs by 48 hpf (Fig. 5 I1, J1, compare to Fig. 3A2, A3). Mmp11-GFP and laminin-111 were found to co-localize at MTJs in *Tg(HSP:mmp11-GFP)* embryos at 26 and 48 hpf (Fig. 5 I1, J1 insets). This result suggests that laminin-111 deposition was unaffected by Mmp11 overexpression. Therefore, laminin-111 regulates Mmp11 localization to MTJs but not vice versa. This suggests that laminin is upstream of Mmp11 in the regulation of Fn. Although our data do not rule out the possibilities of bidirectional or non-linear signaling, they do suggest that laminin-111 functions upstream of Mmp11 in this context.

### Fn levels correlate with phenotypic severity of *laminin* mutants

**Fig. 6:**
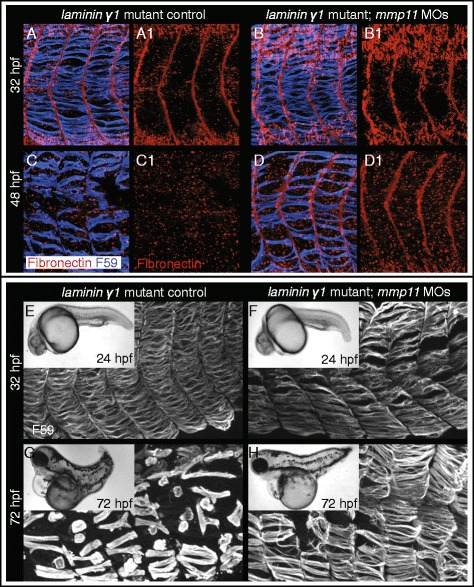
Injection of *mmp11* MOs increases Fn at the 48 hpf MTJ in *laminin gamma1* mutants and improves slow-twitch fiber morphology. **A**–**D** Anterior left, dorsal top, side-mounted, 3D projections of embryos stained for slow-twitch fibers with F59 antibody (*blue*) and Fn antibody (*red*). **A** 32 hpf *laminin gamma1* mutant. **B** 32 hpf *laminin gamma1* mutant; *mmp11* morphant. **C** 48 hpf *laminin gamma1* mutant. **D** 48 hpf *laminin gamma1* mutant; *mmp11* morphant. Note that in 48 hpf *laminin gamma1* mutants, Fn is not clearly concentrated at MTJs (**C**, *n* = 14 embryos). In contrast, Fn robustly concentrates at MTJs in *laminin gamma1* mutants injected with *mmp11* morpholinos at 48 hpf (**D**, *n* = 8 embryos). **E**–**H** Anterior left, dorsal top, side-mounted, 3D projections of embryos stained with anti-F59 antibody. *Small inset boxes* are brightfield, whole mount, live images. **E** *laminin gamma1* mutant at 24 (*inset*) or 32 hpf. **F** *laminin gamma1* mutant injected with *mmp11* MOs at 24 (*inset*) or 32 hpf. Note that overall body shape and slow-twitch muscle fibers are similar in both mutants and mutant morphants at 24 and 32 hpf. **G** *laminin gamma1* mutant at 72 hpf. **H** *laminin gamma1* mutant injected with *mmp11* MOs at 72 hpf. Slow-twitch muscle fiber detachment can be clearly seen in *laminin gamma1* mutant embryos at 72 hpf. However, *laminin gamma1* mutants injected with *mmp11* MOs have less fiber detachment at this time point

We showed that laminin is required for normal Mmp11 expression and Fn levels at the MTJ: in the absence of laminin, Fn levels are increased and Mmp11 levels are decreased. Interestingly, the perdurance of Fn at the MTJ *laminin gamma1* mutants is transient. Increased Fn was clear at 32 hpf (Fig. 6A) but Fn was not observed in mutant MTJs at 48 hpf (Fig. 6C). Injection of *mmp11* MOs into *laminin gamma1* mutants resulted in the expected persistence of Fn at the MTJ at 32 hpf (Fig. 6B) but strikingly, Fn persisted in morphant/mutants even at 48 hpf (Fig. 6D). We next tested whether prolonging the transient increase in Fn at MTJs in *laminin* mutants had any effect on the *laminin* mutant phenotype by injecting *mmp11* MOs into *laminin gamma1* mutant zebrafish. Neither the whole embryo morphology nor the slow-twitch muscle of *laminin* mutants appeared different from mutant morphants at 24 or 32 hpf (Fig. 6E–F). By 72 hpf, slow-twitch muscle fibers had degenerated and could be visualized as spheres instead of cylindrical fibers in *laminin* mutants (Fig. 6G). Slow-twitch muscle fiber degeneration was drastically reduced in *laminin gamma1* mutants injected with *mmp11* MOs compared to mutant controls (Fig. 6H). Thus, Mmp11 knockdown can partially compensate for the muscle defects seen in *laminin gamma1* mutants, likely via prolonging the presence of Fn at the MTJ. These data suggest that, in the absence of laminin, the amount of Fn has a significant effect on the rate and amount of muscle degeneration. All together, we find that myomatrix crosstalk between laminin and Fn regulates Mmp11-dependent remodeling of zebrafish MTJs during development and that this remodeling can be manipulated to improve muscle tissue structure.

## Discussion

**Fig. 7:**
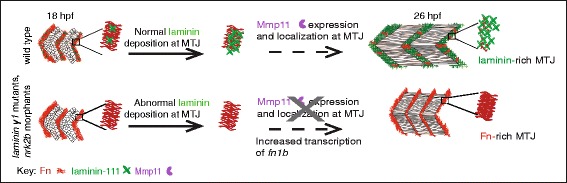
Mechanism of in vivo Fn downregulation at the MTJ. *Upper panel*: normal scenario—laminin is polymerized at the MTJ, Mmp11 expression is localized at the MTJ, and Fn is subsequently downregulated. *Lower panel*: scenario when laminin deposition/organization is disrupted—Mmp11 expression is reduced, Mmp11 localization is abnormal, and Fn perdures at the MTJ

The ECM is an essential component of metazoan tissues and exhibits extraordinary complexity and dynamics at a molecular level. The delicate balance between the degradation and deposition of ECM constituents is constantly changing, and its regulation is central to normal development and tissue homeostasis. Thus, it is not surprising that misregulation of ECM remodeling plays a role in aging and disease. ECM components are regulated on many different levels: regulation of transcription (including alternate splicing and miRNA-mediated silencing), translation, protein trafficking, posttranslational modifications, and formation of multiprotein complexes. Most of what is known about ECM remodeling is based on in vitro studies, and a current challenge is to relate these insights to in vivo processes. Towards this end, we investigated mechanisms of Fn downregulation during MTJ development. We show that one initial cue for Fn downregulation is organized deposition of a different ECM protein, laminin. Laminin downregulates *fn1b* transcription and potentiates Mmp11 localization to the MTJ. Mmp11 is then both necessary and sufficient for Fn downregulation (Fig. 7). Therefore, we have elucidated a novel regulatory pathway that mediates Fn downregulation in vivo.

### Mmp11: a critical regulator of Fn levels in vivo

The matrix metalloproteinases (MMPs) cleave all known ECM proteins. MMPs are tightly regulated during development and homeostasis, and changes in MMP expression occur in many diseases [27, 28]. For example, several MMPs were originally identified because of their upregulation in cancer cell lines. Stromelysin-3 (MMP11 in mammals or Mmp11 in zebrafish) is over expressed in several cancers, where its abundance correlates with tumor aggressiveness and concomitant mortality rates [47–49]. Specifically, increased levels of MMP11 in human and mouse breast cancers are associated with increased metastasis and poor patient prognosis [49–53]. Multiple lines of evidence suggest that MMP11 is a protein with important functions in normal development as well as disease. MMP11 expression is found in remodeling ECM and is required for cell migration during normal morphogenesis in *Xenopus* [54]. Here, we show that (1) Mmp11 protein localizes to the MTJ during myogenesis in a laminin-dependent manner, (2) Mmp11 is necessary and sufficient for Fn downregulation at the MTJ, and (3) inhibiting Mmp11 activity in *laminin* mutants increases Fn levels and improves muscle structure. Thus, our data extend and support previous experiments indicating that Mmp11 plays crucial roles in morphogenesis.

The data shown here do not resolve whether Mmp11 degrades Fn in vivo directly or indirectly due to technical limitations. MMP11 shows weak protease activity against Fn in vitro [46]. However, it remains largely undetermined whether the ability or inability of MMPs to cleave substrates in vitro corresponds with their in vivo activities. It is possible that MMP11 cleaves Fn in vivo more efficiently, perhaps due to mechanical load on the Fn fibrils, contractile forces, or other interactions that position scissile bonds such that they become accessible to the protease. It is also possible that the effects of MMP11 on Fn are indirect. MMP11 is activated intracellularly by Furin [55] and is capable of cleaving and activating both extracellular and intracellular proteins, such as proMMPs [56]. Therefore, MMP11 may have indirect effects on Fn downregulation by activating other proteases. Recently, it has been shown that Mmp14a is expressed at the zebrafish MTJ at 24 and 48 hpf [57], which makes it a good candidate to be involved in the regulation of Fn. MMP14 is known to degrade Fn [58] and is critical for myoblast differentiation in vitro [59]. In addition, MMP11 is a MMP14 substrate and MMP14 thereby negatively regulates MMP11 [60]. Interactions between MMP11, MMP14, laminin, and Fn will be interesting to identify in future studies. Alternatively, MMP11 could affect Fn levels indirectly via MMP11-dependent regulation of receptors for laminin. MMP11 cleaves the 67kD non-integrin laminin receptor (LR) in vivo [61, 62]. It is possible that MMP11 does not directly degrade Fn but rather releases LR-mediated inhibition of some other protease. Regardless, our data clearly show that Mmp11 is necessary and sufficient for Fn downregulation in vivo. It will be interesting in the future to identify molecular mechanisms underlying this ECM remodeling, such as the mechanisms of Mmp11 action and the effect of ectopic Mmp11 in disease states associated with excess Fn (i.e., fibrosis).

### Fn levels are dynamically regulated in development and disease

Fn plays important roles in development, repair/regeneration, and the transition to disease states in many organs. Fn is required for mesoderm specification, left-right patterning, trunk elongation, angiogenesis, heart morphogenesis, and migration of myocardial cells [21, 63–69]. In skeletal muscle, Fn is required for muscle development [22, 42, 70] and is both necessary and sufficient in the stem cell niche for muscle regeneration [5]. However, upregulation of Fn, while critical for certain developmental processes, is also linked to tumor metastasis and fibrosis. Fn is upregulated in many cancers and promotes formation of the premetastatic niche, which generates a microenvironment more hospitable for tumor cell adhesion and proliferation [71–74]. Fibrosis, the aberrant deposition of Fn and various collagens, diminishes organ function. Inhibiting Fn deposition not only prevents but can reverse liver fibrosis, possibly via both reducing cell infiltration and not providing a scaffold for collagen deposition [75]. Fibrosis occurs in skeletal muscle during aging, atrophic conditions, and disease. Fn is a serum biomarker for Duchenne muscular dystrophy, suggesting that excess Fn contributes to muscle pathology [76]. Thus, the importance of precise Fn regulation for skeletal muscle development, regeneration, and function is mirrored in other organ systems. While these multifactorial roles for Fn demonstrate the importance of dynamically regulating Fn levels, the molecular mechanisms regulating Fn expression and polymerization are just beginning to be elucidated and exceedingly little is known about how Fn is degraded in vivo. Fn downregulation during development is a normal part of the transition to a laminin-rich basement membrane at the myotendinous junction (MTJ). Our data show that laminin, via subcellular localization of Mmp11 and inhibition of *fn1b* transcription, is necessary for Fn downregulation. While likely an indirect effect of Mmp11, our data implicate Mmp11 and laminin in a novel network that regulates Fn downregulation in vivo. As Fn is dynamically regulated in other systems, such as its degradation behind migrating myocardial cells [21], it will be interesting to determine if there is a similar regulation of Fn by laminin in other developmental, cell migratory, and/or disease contexts.

### Crosstalk between myomatrix proteins and cellular adaptation in development and disease

Our data provide compelling evidence that there is crosstalk between the ECM proteins Fn and laminin during muscle development and that this crosstalk results in cellular adaptations that protect muscle structure when development is disrupted. We previously showed an inverse expression pattern of Fn and laminin during MTJ development: Fn is downregulated and laminin is polymerized adjacent to fast-twitch muscle fibers as they elongate and attach to the MTJ [9]. Here, we show that laminin deposition/organization acts as a “checkpoint” for Fn downregulation. To our knowledge, this is an unrecognized interaction between Fn and laminin and represents a novel paradigm in which laminin is permissive for Fn downregulation. In a developmental context, such a mechanism ensures that an established matrix is continuously present, while simultaneously permitting biochemical and structural remodeling.

Crosstalk between ECM molecules Fn and Collagen1 has been shown to regulate MMP expression in vitro. Exogenous Fn caused human fibroblasts to upregulate MMP15 and MMP9, and subsequent addition of Collagen1 to the Fn-rich matrix attenuated this response [77]. Thus, there appears to be crosstalk between these ECM molecules as well as between these ECM molecules and enzymes that remodel ECM. It will be an interesting line of future investigation to look at such interactions in vivo as well as to elucidate roles for collagen molecules in normal and pathological remodeling of the myomatrix.

The crosstalk between laminin and Fn presented here is reminiscent of the crosstalk between receptors for ECM proteins that occurs and can be beneficial in muscle diseases. Adhesion of muscle fibers to the laminin-rich basement membrane (BM) is critical for muscle development and homeostasis. Many muscular dystrophies, including Duchenne, Becker, and Congenital muscular dystrophy with integrin alpha7 deficiency, result from mutations that disrupt adhesion of muscle fibers to their BM. There are two main receptor complexes that anchor muscle cells to the BM: the dystrophin-glycoprotein complex (DGC) and integrin-based adhesions. Although both of these receptors have unique functions [78], they can partially compensate for each other in adhesion to laminin. The fact that patients with Duchenne muscular dystrophy (DMD), which affects a member of the DGC, show increased expression of integrin alpha7 [79], highlights the intrinsic crosstalk between cell-ECM adhesion complexes that occurs in diseased muscle.

Cell-ECM adhesion complexes sense multiple types of physiological changes and interface with every major signaling pathway. Thus, cell-ECM adhesion complexes are ideally situated to facilitate appropriate responses to physiological change. One approach towards elucidating the roles of cell-ECM crosstalk in cellular adaptation is to investigate the dynamic regulation of cell-ECM adhesion during embryonic development. We focus on zebrafish MTJ development as a model of in vivo cell-ECM adhesion and cell adaptation because muscle tissue is strikingly adaptable. While the increased Fn at the MTJ of *laminin gamma1* mutants did not completely rescue muscle morphogenesis in these mutants, just as the increased integrin alpha7 expression observed in humans with DMD does not prevent muscle atrophy, one key experiment shows that reducing Fn downregulation in *laminin* mutants did have a positive effect on slow-twitch muscle fibers. Injection of *mmp11* MOs into *laminin gamma1* mutants increased Fn abundance and decreased slow-twitch muscle degeneration. These data support the hypothesis that reciprocal regulation of ECM proteins plays a role in cell adaptation in adverse conditions (such as the absence of laminin-111 in *laminin gamma1* mutants) and suggests that this line of investigation (elucidating mechanisms of cell-ECM adhesion that regulate cell adaptation during development) can potentially be translated into meaningful therapies for certain diseases.

## Conclusions

We have addressed the regulatory mechanism of Fibronectin downregulation during MTJ development in vivo. Our data show that normal laminin organization acts as a “checkpoint” for Fn downregulation. Furthermore, laminin signaling modulates both the expression of *fn1b* and localization of Mmp11 to MTJs. The augmentation of Fn at MTJs in *mmp11* morphants was reversed by driving overexpression of Mmp11-GFP, demonstrating that Mmp11 is both necessary and sufficient for Fn downregulation (and the specificity of our morpholinos). Overall, we identify a new mechanism in the myomatrix that regulates the remodeling process during morphogenesis in vivo, and that may be profitably targeted in the many pathological conditions in which Fn is dysregulated, to improve muscle tissue structure.

## Abbreviations

BM: basement membrane
BSA: bovine serum albumin
DGC: dystrophin-glycoprotein complex
DMD: Duchenne muscular dystrophy
ECM: extracellular matrix
ERM: embryo rearing media
Fn: Fibronectin
hpf: hours post fertilization
Hr: hour
HSP: heat shock promoter
LR: laminin receptor
Mmp: matrix metalloproteinase
MO: morpholino
MP: muscle pioneer
MTJ: myotendinous junction
Nrk2b: nicotinamide riboside kinase 2b
PBST: phosphate buffered saline with 0.1 % Tween20
PFA: paraformaldehyde
RT: room temperature
Tg: transgenic
tTg: transient transgenic
